# Early Outcomes of Carotid Revascularization in Retrospective Case Series

**DOI:** 10.3390/jcm10050935

**Published:** 2021-03-01

**Authors:** Petroula Nana, George Kouvelos, Alexandros Brotis, Konstantinos Spanos, Efthimios Dardiotis, Miltiadis Matsagkas, Athanasios Giannoukas

**Affiliations:** 1 Vascular Surgery Department, Larissa University Hospital, Faculty of Medicine, School of Health Sciences, University of Thessaly, 41334 Larissa, Greece; petr.nana7@hotmail.com (P.N.); spanos.kon@gmail.com (K.S.); milmats@gmail.com (M.M.); agiannoukas@hotmail.com (A.G.); 2 Department of Neurosurgery, Larissa University Hospital, Faculty of Medicine, School of Health Sciences, University of Thessaly, 38221 Volos, Greece; alexgbrodis@yahoo.com; 3 Department of Neurology, University Hospital of Larissa, School of Medicine, University of Thessaly, 38221 Volos, Greece; edar@med.uth.gr

**Keywords:** carotid revascularization, carotid angioplasty, stent, carotid endarterectomy

## Abstract

Background: Most data in carotid stenosis treatment arise from randomized control trials (RCTs) and cohort studies. The aim of this meta-analysis was to compare 30-day outcomes in real-world practice from centers providing both modalities. Methods: A data search of the English literature was conducted, using PubMed, EMBASE and CENTRAL databases, until December 2019, using the Preferred Reporting Items for Systematic Reviews and Meta-Analysis statement (PRISMA) guidelines. Only studies reporting on 30-day outcomes from centers, where both techniques were performed, were eligible for this analysis. Results: In total, 15 articles were included (16,043 patients). Of the patients, 68.1% were asymptomatic. Carotid artery stenting (CAS) did not differ from carotid endarterectomy (CEA) in terms of stroke (odds ratio (OR) 0.98; 0.77–1.25; *I*^2^ = 0%), myocardial ischemic events (OR 1.03; 0.72–1.48; *I*^2^ = 0%) and all events (OR 1.0; 0.82–1.21; *I*^2^ = 0%). Pooled stroke incidence in asymptomatic patients was 1% (95% CI: 0–2%) for CEA and 1% for CAS (95% CI: 0–2%). Pooled stroke rate in symptomatic patients was 3% (95% CI: 1–4%) for CEA and 3% (95% CI: 1–4%) for CAS. The two techniques did not differ in either outcome both in asymptomatic and symptomatic patients. Conclusion: Carotid revascularization, performed in centers providing both CAS and CEA, is safe and effective. Both techniques did not differ in terms of post-procedural neurological and cardiac events, both in asymptomatic and symptomatic patients. These findings reiterate the importance of a tailored therapeutic strategy and that “real-world” outcomes may only be valid from centers providing both treatments.

## 1. Introduction

As stroke is one of the main disabling and fatal causes in the developed world, carotid atherosclerosis management enforces its role in daily clinical practice [1,2]. The number of strokes will increase from 1.1 million per year in 2000 to more than 1.5 million per year in the next five years [2]. The beneficial role of carotid endarterectomy (CEA) in preventing stroke, mostly to symptomatic and to a lesser extent to asymptomatic patients, has been well established [1]. In the era of less invasive procedures, carotid artery stenting (CAS) has emerged as an alternative therapeutic modality. Current guidelines recommend CEA as the standard treatment in symptomatic and asymptomatic patients, while CAS is suggested as an alternative approach in high-risk patients with adverse medical co-morbidities or anatomical restrictions [1].

The risk of peri-procedural events, including stroke, myocardial infarction (MI) and death, is reasonable to consider in the decision making. CAS offers comparable survival as CEA, while the risk of stroke remains higher in CAS, and of MI in CEA [3]. Although randomized controlled trials (RCTs) of the previous decade are essential for providing level I evidence, and current recommendations have been extracted based on them, the results from “real-world” practice do not always reflect the outcomes reported in the RCTs. In daily clinical practice, the experience is not balanced, as in many cases the reports come from centers providing only one of the treatment modalities; thus, patient selection and reporting biases are inevitable.

In order to assess the 30-day outcomes of both CEA and CAS in “real-world” practice, we conducted the present meta-analysis including only studies from centers providing both treatment modalities.

## 2. Methods

### 2.1. Eligibility Criteria

The aim of the present meta-analysis was to investigate real-world evidence in carotid artery disease treatment. Real-world evidence means evidence obtained from real-world data (RWD), which are observational data obtained outside the context of RCTs and generated during routine clinical practice. The objectives, methodology of the systematic review, analysis and inclusion criteria for study enrollment were pre-specified and registered to the PROSPERO (CRD 42020153756) [4]. The present meta-analysis was conducted using the Preferred Reporting Items for Systematic Reviews and Meta-Analysis statement (PRISMA) guidelines [5]. Data extraction was performed by two independent reviewers (P.N., G.K.) using a non-blinded standardized form, and any discrepancies were resolved by consulting a third reviewer (K.S.). No informed consent or institutional review board approval was required, as no patients were used for the conduction of this analysis. Studies considered for inclusion and full-text review fulfilled the following criteria: (1) to report on patients that underwent carotid revascularization for atherosclerotic carotid stenosis, (2) to provide 30-day outcomes as new neurological events, myocardial ischemic events and/or death, (3) to present outcomes of both CAS and CEA techniques, and (4) to report experience from single centers providing both treatment modalities. Randomized controlled trials; data from national registries; studies reporting on less than 50 cases, without follow-up outcomes; technical characteristics different from transcarotid stenting; and studies providing outcomes other than the aforementioned were excluded. The primary selection was based on title and abstract and a secondary scrutiny on the full text.

### 2.2. Search Strategy

A data search of the English medical literature was conducted using PubMed, EMBASE and CENTRAL databases, until 31 December 2019. The P.I.C.O. (patient; intervention; comparison; outcome) model was used to define the clinical questions and select relevant articles (Supplementary Table S1) [6]. The following search terms including Expanded Medical Subject Headings (MeSH) were used in various combinations: ‘‘carotid stent”, “carotid angioplasty”, “carotid endarterectomy”, “cohort studies”, “comparative study”.

### 2.3. Data Extraction and Quality Assessment

A standardized data extraction Microsoft Excel file was developed. Data were retrieved from the text or tables. Extracted data included study characteristics such as author, date of publication and study nature. Furthermore, the following clinical information was collected: baseline demographics (age, sex), symptomatic or asymptomatic carotid disease, post-operative neurological events (including stroke and transient ischemic attack), myocardial ischemic events and death, as well as the composite events (neurological event/myocardial ischemia/death) during the early follow-up. Individual studies were assessed for clarity of reporting using the Robins I tool for non-randomized studies (Supplementary Table S2) [7]. The Grading of Recommendations Assessment, Development and Evaluation (GRADE) approach was used to evaluate the quality of evidence assessment and the summary of findings for each of the included outcomes, to ensure that the effectuated judgments are systematic and transparent [8]. Supplemental Table S3 summarizes the results of the evidence quality assessment using the GRADE approach, while Supplementary Table S4 represents a summary of the evidence. Neurological events included stroke (major and minor) and transient ischemic attack during the 30-day post-operative period. Death included all-cause mortality during the same period.

### 2.4. Statistical Analysis

The outcomes were summarized as proportion incidence and odds ratio along with their 95% confidence intervals (CIs), through a proportion meta-analysis and a paired meta-analysis, respectively. The inter-study heterogeneity was evaluated using the significance of the Cochran’s Q-metric (*p*Q) and quantified by the Higgins *I*² statistics. Significance was set at *p* < 0.05, and we used continuity correction equal to 0.5 for metrics associated with zero events. The pooled estimate was assessed using the random effects model in the presence of inter-study heterogeneity (*I*² > 50%), or else with the fixed effects model. Publication bias was eyeballed by trim-and-fill funnel plots. All statistical analyses were executed using the packages “meta” and “metaphor” of the R-statistical environment [9,10].

## 3. Results

**Figure 1:**
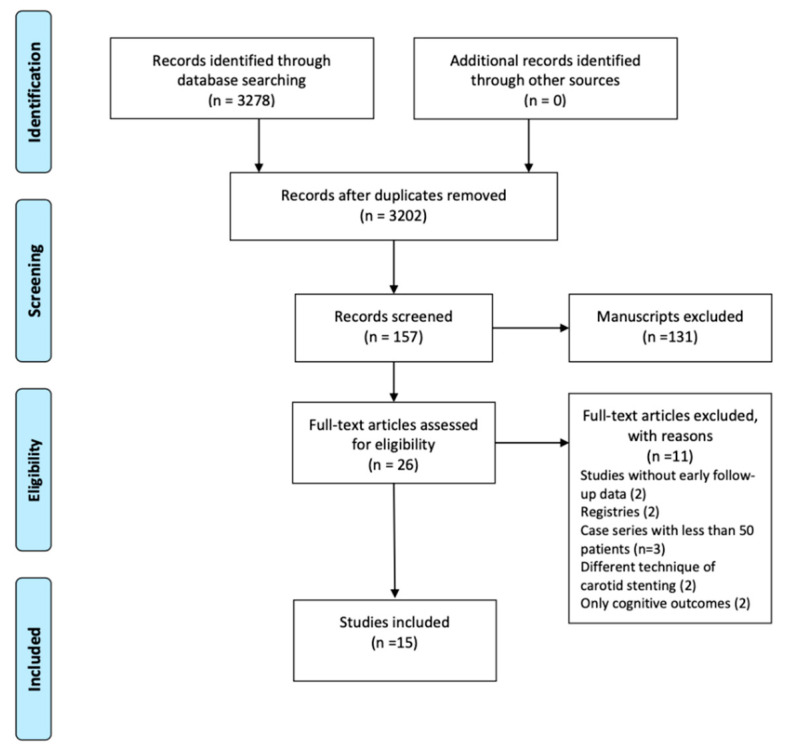
The present meta-analysis was conducted using the PRISMA guidelines and included 15 articles with 16,043 patients.

The initial search identified 3252 articles potentially suitable for inclusion. After exclusion of articles whose titles had no relevance to the topic, the full texts of 26 articles were retrieved and assessed for eligibility. The final analysis included 15 articles published between 2003 and 2019, which included a total of 16,043 patients (Figure 1). The study cohorts ranged from 65 to 6940 patients. All articles presented the results of observational, retrospective, comparative studies [11,12,13,14,15,16,17,18,19,20,21,22,23,24,25]. The studies’ characteristics are shown in Table 1. In nine studies the operators were vascular surgeons exclusively, in three studies vascular surgeons and radiologists, in two studies vascular surgeons and cardiologists, while in one study CAS was performed by cardiologists, radiologists and neurosurgeons. Only four studies reported on long-term follow-up outcome (mean follow-up 24, 65, 120, 39.6 months, respectively).

Patients underwent carotid revascularization using CAS or CEA for symptomatic or asymptomatic carotid disease. Males were 72.7%, and the mean age was estimated at 71.5 years (range 64–86.9 years), with a mean age at 69.7 years in the CAS and 69.5 years in the CEA group. Coronary artery disease was the most common comorbidity among CEA 84% (95% CI 78–88%) and CAS 85% (95% CI 79–90%) patients, respectively. The distribution of comorbidities is presented in Table 2. There were no significant differences between the two groups in either comorbidity factor.

Asymptomatic patients represented 68.1% of the whole cohort. The distribution of asymptomatic carotid disease was higher in both groups (79.8% in CAS and 64.8% in CEA). In CAS, embolic protection was used in 3911 patients (90.1%), while in four studies the use of a filter or other protection device was not recorded [11,19,22,25].

### 3.1. Synthesis of Results and Outcome

**Figure 2:**
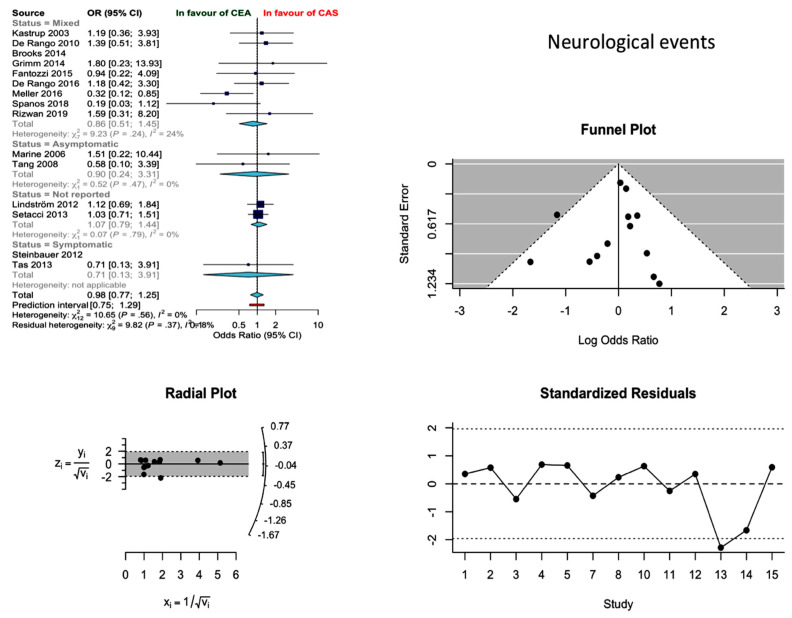
Differences in neurological event rate and related funnel plot between the CEA and CAS groups [11,12,13,14,15,16,17,18,19,20,21,22,23,24,25].

Thirteen studies reported on the occurrence of neurological events during the 30-day postoperative period, resulting in an estimated pooled proportion incidence of 2.4% (95% CI: 1.69–3.4%) and 2.75% (2.01–3.76%) for CEA and CAS, respectively. The two techniques did not differ in terms of post-procedural new neurological events (odds ratio (OR) 0.98; 0.77–1.25; *I*^2^ = 0%). The results were robust to major changes (Q 0.69; df 3; *p* 0.841) after the stratification of the evidence according to the preoperative neurological status (asymptomatic vs. symptomatic). Meanwhile, the relevant funnel plot was not indicative of significant publication bias (Figure 2).

**Figure 3:**
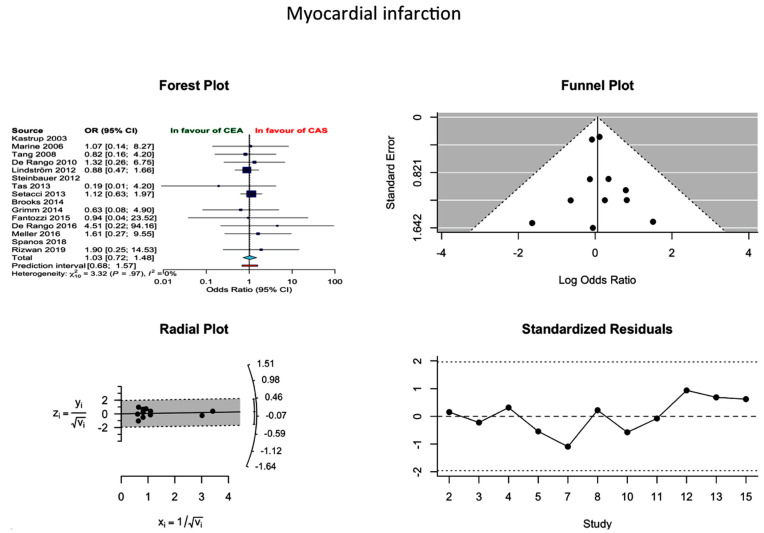
Differences in MI rate and related funnel plot between the CEA and CAS groups [11,12,13,14,15,16,17,18,19,20,21,22,23,24,25].

Based on 13 studies, the pooled proportion incidence of MI after CEA and CAS was 1.66% (1.44–1.93%) and 1.16% (0.08–1.55%), respectively. In the absence of publication bias, the difference between the two approaches was not statistically significant (OR 1.03; 0.72–1.25; *I*^2^ = 0%) (Figure 3).

**Figure 4:**
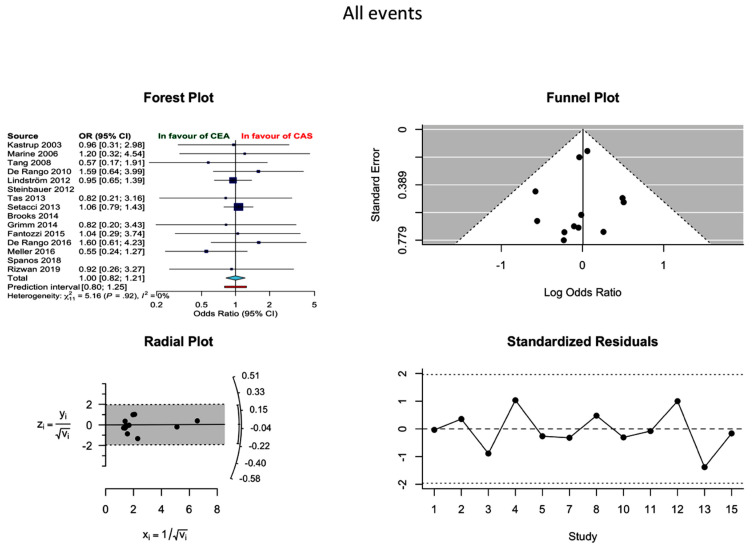
Differences in all events rate and related funnel plot between the CEA and CAS groups [11,12,13,14,15,16,17,18,19,20,21,22,23,24,25].

Incidence of all events (neurological events/MI/death) was reported in twelve studies, without establishing a statistical difference between the two modalities (OR 1.0 0.82–1.21, *I*^2^ = 0%). More specifically, the proportion of incidences of all events in CEA and CAS were 3.96 (2.95–5.30) and 4.23 (3.11–5.74), respectively (Figure 4). The probability of publication bias was low.

### 3.2. Asymptomatic Patients

**Figure 5:**
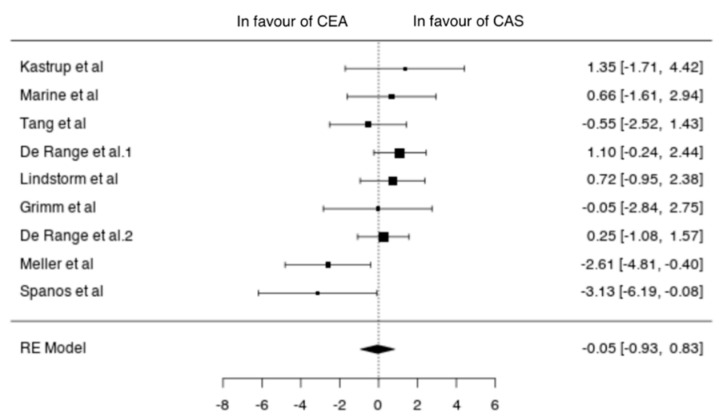
Differences in neurological events between the CEA and CAS groups in asymptomatic patients [11,12,13,14,15,20,22,23,24].

Data on asymptomatic patients were available from nine studies (2850 patients; 1707 CEA, 1143 CAS) [11,12,13,14,15,20,22,23,24]. In asymptomatic patients undergoing CEA the pooled incidence of neurological event, MI and death was 1% (95% CI 0–2%), 0% (95% CI 0–1%) and 0% (95% CI 0–1%), respectively. The pooled incidence of neurological events, MI and death after CAS was 1% (95% CI 0–2%), 1% (95% CI 0–1%) and 1% (95% CI 0–1%), respectively. In asymptomatic patients the two techniques did not differ in terms of neurological events (OR −0.05 (−0.93, 0.83), *p* = 0.907), MI (OR −0.85 (−2.07, 0.38), *p* = 0.177) and death (−0.61 (−1.59, 0.36), *p* = 0.218) (Figure 5).

### 3.3. Symptomatic Patients

**Figure 6:**
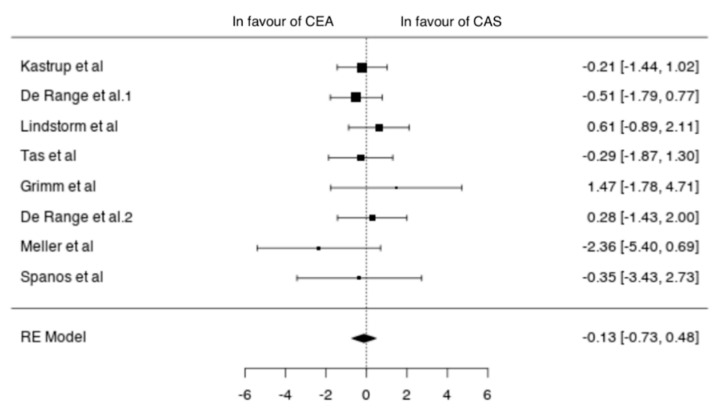
Differences in neurological events between the CEA and CAS groups in symptomatic patients [11,12,13,14,15,20,22,23,24].

Eight studies reported outcomes in symptomatic patients (1671 patients; 1151 CEA, 520 CAS) [11,14,15,17,20,22,23,24]. In symptomatic patients undergoing CEA the pooled incidence of neurological events, MI and death was 3% (95% CI 1–4%), 0% (95% CI 0–1%) and 1% (95% CI 0–1%), respectively. The pooled incidence of neurological events, MI and death after CAS was 3% (95% CI 1–4%), 1% (95% CI 0–1%) and 1% (95% CI 0–2%), respectively. In symptomatic patients the two techniques showed no differences in terms of neurological events (OR −0.13 (−0.73, 0.48), *p* = 0.681), MI (OR −0.03 (−1.38, 1.33), *p* = 0.968) and death (−0.18 (−1.17, 0.81), *p* = 0.721) (Figure 6).

## 4. Discussion

Several RCTs comparing CEA versus CAS have demonstrated that CEA remains the standard of care in stroke prevention in symptomatic and asymptomatic patients with high-grade carotid stenosis [1,26,27,28]. However, as CAS has evolved through years, recent reports have shown that both techniques may be used with comparable outcomes, after a detailed evaluation of carotid plaque characteristics and patients’ specific risk factors [29]. The present meta-analysis has demonstrated that among centers providing both CEA and CAS, no differences were found in 30-day outcomes. The incidence of 30-day neurologic events presented a tendency in favor of CEA (CEA: 2.1% and CAS: 2.6%). However, CAS-related neurologic events were lower than those reported in RCTs. In CREST (Carotid Revascularization Endarterectomy vs. Stenting Trial), which had the most austere enrollment protocol, the periprocedural stroke rate was 4.1% for the CAS and 2.3% for the CEA group [30]. In the rest of the RCTs, EVA-3S (Endarterectomy versus Angioplasty in Patients with Symptomatic Severe Carotid Stenosis) was stopped prematurely for safety and futility reasons, as the 30-day incidence of any stroke or death was 3.9% after CEA vs 9.6% after CAS, while SPACE trial (Stent-Protected Angioplasty versus Carotid Endarterectomy) failed to prove the non-inferiority of CAS compared to CEA in terms of periprocedural complication rate [26,27]. Along this line, ICSS (International Carotid Stenting Study) concluded that CEA should remain the treatment of choice due to a 4.0% of disabling stroke or death event rate in CAS versus 3.2% in CEA [28]. Furthermore, all events (stroke/death/MI) were significantly higher in CAS in this trial [28].

RCTs have been subject to criticism because of their strict inclusion and exclusion for patients’ and centers’ eligibility criteria, raising concerns whether their results could be extrapolated to the daily clinical practice [31]. In RCTs, by using inclusion and exclusion (“high-risk”) criteria, a significant portion of eligible carotid patients were eventually excluded. Each technique is associated with specific high-risk features. CEA can be considered as high-risk in patients with severe cardiac comorbidity, while plaque morphology (type IV, V and VI) and unfavorable vessel anatomy can also influence the outcomes of CAS. A retrospective analysis has shown that aortic arch anatomy and a careful pre-operative imaging assessment is very important for the prevention of embolic events during catheter manipulations in the aortic lumen [32]. Therefore, some patients may be more suitable candidates for CEA but not for CAS and vice versa [31]. This strategy of an individualized treatment selection can only be offered in centers with adequate experience in both CEA and CAS, and this is the reason why, in this systematic review, only such experience was included in the analysis.

Detailed evaluation and careful patient selection, operative planning and technical details in the pre- and intra-operative setting seem to be mandatory in improving outcomes for both techniques [31]. However, patients’ risk assessment during pre-operative risk stratification would be incomplete without a thoughtful assessment of interventionists’ experience. Poor outcomes were detected in the initial experience with CAS [33,34], and rationally the learning curve of each interventionist and each center providing CAS has affected its outcomes. At the same time, technological innovations related to stent design and cerebral protection methods, surgeon credentialing and rigorous training through recent years have inevitably affected the efficacy of CAS contributing to the improved reported outcomes [31]. Operators participating in the most recent RCTs had to perform a specific number of procedures to prove their expertise before being allowed to participate in the trial. In most RCTs, the interventionists’ experience needed to enroll patients was limited to less than 25 CAS cases [30,35]. Furthermore, the majority of RCTs conducted during the previous decade were inherent to such influence of the limited experience in CAS. In the current systematic review, the majority of the studies have included many patients treated during the last decade when the endovascular treatment had rapidly evolved and many of the current technological advancements have been already adopted. Unfortunately, the effect of CAS evolution on patients’ outcomes cannot be proved in the current analysis, as most studies have a wide time period spanning an average of seven years.

RCTs have shown significant differences between CEA and CAS in symptomatic patients. European Society for Vascular Surgery (ESVS) guidelines recommend a potential role for both CEA and CAS, but the levels of evidence are slightly lower for CAS than for CEA, mostly because 30-day risks of death/stroke in the RCTs were significantly higher after CAS than after CEA [1,36]. Furthermore, in a systematic review, Paraskevas et al. found that 13/18 administrative dataset registries (72%) reported 30-day death/stroke rates in excess of the recommended 6% risk threshold following CAS in symptomatic patients, while 5/18 (28%) reported stroke rates in excess of 10% [37]. On the contrary, only 1/18 registries reported 30-day death/stroke rates exceeding 6% in patients undergoing CEA [37]. In this analysis, no difference in a 30-day outcome was found in symptomatic patients treated with either modality, while stroke rate for both CEA and CAS was 3%. This finding is creating concerns that the results obtained in RCTs and registries may not be generalizable into routine clinical practice.

For asymptomatic patients, levels of evidence are also slightly less for CAS than for CEA [1]. Thirty-day risks of death/stroke in the largest RCTs, which used experienced CAS interventionists, were only just within the accepted 3% risk threshold [38,39]. In the same systematic review, Paraskevas et al. found that 9/21 administrative dataset registries (43%) reported 30-day death/stroke rates in excess of the recommended 3% risk threshold after CAS in asymptomatic patients, while 7/21 (33%) reported stroke rates in excess of 4% [37]. On the contrary, only 1/21 registries reported 30-day death/stroke rates exceeding 3% in patients undergoing CEA. Data for asymptomatic patients from centers providing both CEA and CAS are different. In the present analysis, stroke rates for both treatment modalities were 1% in asymptomatic patients. Those rates are lower than those reported in RCTs and registries, showing that results can be improved when a tailored therapeutic strategy is followed.

CEA and CAS may be performed not only by vascular surgeons but also by neurosurgeons, cardiologists or radiologists. Each specialty has unique education and capabilities. Meller et al. compared the 30-day CAS results between the three different specialties and found the interventional cardiology group demonstrated the lowest rates of the composite endpoint (2.8%), and the interventional radiology group the highest (12.1%) [23]. Although this analysis may be biased by significant selection criteria, as the interventional cardiologists performed the vast majority of the CAS procedures and had larger experience, we should admit that profound logistics and the requirement of a multidisciplinary approach of related specialties should be fulfilled for a hospital to be able to provide both procedures with equal results.

RCTs’ results do not guide management in all patients with carotid artery stenosis in daily clinical practice. It is not always suitable to generalize the results of cautiously conducted RCTs with specific inclusion and exclusion criteria to the general population and in all centers, regardless of their experience. In the present analysis we included centers with significant experience in both procedures, where each patient’s treatment could be tailored according to specific indications and anatomic characteristics. Certain centers of excellence may accomplish better results than those reported in RCTs, whereas other less experienced institutions may achieve significantly worse outcomes. These innate weaknesses limit the generalizability of the results of RCTs to every individual patient encountered in everyday clinical practice. Additionally, it is rational that outside RCTs, in every center there might be a number of physicians with different levels of expertise; thus, each could perform the procedure that is more familiar to them, while in the RCTs the physicians that were included performed both procedures. That is the idea behind an expertise-based approach to trial design, where health professionals only deliver an intervention in which they have expertise, and this has been proposed as an alternative [40].

The aim of the present analysis is not to directly compare RCTs’ outcomes versus single-center studies’ outcomes but to highlight the discrepancy between the accumulated results. Should we rely solely on RCTs because they are less exposed to bias, or should we seek for more real-world evidence as it might deliver some of the information we really need to guide decision strategy in daily practice? The future may not be about RCTs vs. real-world evidence but about RCTs and real-world evidence—not just for the assessment of safety but also of efficacy. Whereas, fully recognizing the need to improve the feasibility of RCTs, we need to explore methods of synthesizing randomized and nonrandomized data.

### Limitations

This systematic review included data across cohort studies to estimate the 30-day outcomes of CAS and CEA in real-world experience. Centers providing both techniques were selected in order to eliminate patient selection and reporting biases. The main limitation of this review is that all studies were of a retrospective nature. The methodological quality of them varied considerably. Furthermore, in technical terms, specific patient selection criteria, type of stent or embolic protection device used, as well as the morphological characteristics of the carotid plaque (synthesis, location and length) were not available in all studies. Observational studies, even from centers with large experience on carotid artery disease treatment, usually lack a systematic, independent neurological examination due to their retrospective nature. Under-reporting of minor neurologic events or chemical MIs may become evident, although those usually do not a have a significant clinical impact on patients’ post-operative course. Although transient neurologic symptoms and subclinical MIs may become underreported, in well-conducted retrospective studies from experienced centers the report of robust endpoints as major stroke, MI with clinical symptoms or biochemical and electrocardiographic markers and deaths remain valid. Furthermore, although this study focusses on 30-day outcomes after CEA/CAS, data on long-term follow up are lacking in the studies included; therefore, the effect of both procedures on late complications could not be assessed.

## 5. Conclusions

Carotid revascularization performed in centers providing both CAS and CEA is safe and effective. Both techniques did not differ in terms of post-procedural neurological and cardiac events, both in asymptomatic and symptomatic patients. These findings reiterate the importance of a tailored therapeutic strategy and that “real-world” outcomes may only be valid from centers providing both treatments.

## Figures and Tables

**Table 1:** The studies’ characteristics.

References	Period	Specialties	Patients	Male (N, %)	Age (Median or Mean + _SD)	Symptomatic (N, %)	CEA	CAS
Kastrup et al. [11]	1999–2001	VS, IR	242	180 (74.3)	70	155 (64)	142	100
Marine et al. [12]	2003–2005	VS	248	148 (59.7)	ΝA	0 (0)	145	93
Tang et al. [13]	2001–2006	VS	326	202 (61.9)	71	0 (0)	206	120
De Rango et al. [14]	2004–2009	VS	567	0 (0)	71	152 (26.7)	325	306
Lindström et al. [15]	2004–2011	VS, IR	6940	NA	ΝA	NA	6474	466
Steinbauer et al. [16]	1999–2002	VS, IR	87	NA	68.5 ± 7.9	87 (100)	44	43
Tas et al. [17]	2011–2012	VS, IC	65	51 (78.5)	ΝA	65 (100)	32	33
Setacci et al. [18]	2000–2010	VS	4638	4005 (86.4)	73.8	NA	2453	2628
Brooks et al. [19]	1998–2002	VS, IC	189	NA	ΝA	104 (55)	94	95
Grimm et al. [20]	2005–2012	VS	182	104 (57.1)	ΝA	55 (30.2)	88	94
Fantozzi et al. [21]	2002–2013	VS	166	93 (56)	86.9	35 (21)	45	129
De Rango et al. [22]	2001–2009	VS	949	670 (70.6)	64	282 (29.7)	500	449
Meller et al. [23]	2007–2013	CAS: IC, IR, NR	718	452 (62.9)	72	270 (37.6)	525	193
Spanos et al. [24]	2006–2016	VS	413	333 (80.6)	69 ± 7.6	135 (32.7)	346	67
Rizwan et al. [25]	2005–2017	VS	313	184 (58.8)	ΝA	110 (35.1)	147	166

SD: standard deviation, CEA: carotid endarterectomy, CAS: carotid artery stenting, NA: not available, VS: vascular surgeons, IC: interventional cardiologists, IR: interventional radiologists, NR: neurosurgeons.

**Table 2:** Patients’ comorbidities in each group.

Studies	CEA				CAS			
	Smoke	HT	DLP	DM	Smoke	HT	DLP	DM
Kastrup et al. [11]	42 (29.6)	118 (83)	63 (44.4)	46 (32.4)	30 (30)	86 (86)	48 (48)	26 (26)
Marine et al. [12]	79 (54.5)	112 (77.2)	97 (66.9)	44 (30.3)	54 (58.1)	85 (91.4)	70 (75.3)	35 (37.6)
Tang et al. [13]	53 (25.7)	169 (82)	138 (67)	60 (29.1)	19	106 (88.3)	84 (70)	38 (31.6)
De Rango et al. [14]	NA	271 (83.4)	178 (54.8)	94 (28.9)	NA	264 (86.3)	196 (64.1)	87 (28.4)
Lindström et al. [15].	NA	NA	NA	NA	147 (31.5)	342 (73.4)	NA	122 (26.2)
Steinbauer et al. [16]	28 (63.6)	34 (77.3)	23 (52.3)	15 (34.1)	19 (44.2)	34 (79.1)	22 (50)	19 (44.2)
Tas et al. [17]	25 (78.1)	21 (65.6)	NA	20 (62.5)	20 (60.6)	25 (75.8)	NA	11 (33.3)
Setacci et al. [18]	1298 (28)	1763 (38.01)	641 (13.8)	832 (17.9)	1415 (53.8)	1653 (62.9)	723 (27.5)	954 (36.3)
Brooks et al. [19]	NA	NA	NA	NA	NA	NA	NA	NA
Grimm et al. [20]	NA	83 (94.3)	85 (96.5)	36 (40.9)	NA	83 (88.3)	72 (76.6)	27 (28.7)
Fantozzi et al. [21]	NA	NA	NA	NA	NA	NA	NA	NA
De Rango et al. [22]	NA	370 (74)	301 (60.2)	156 (31.2)	NA	373 (83.1)	308 (68.6)	151 (33.6)
Meller et al. [23]	257 (49)	474 (90.3)	430 (81.9)	168 (32)	109 (56.5)	182 (94.3)	172 (89.1)	83 (43)
Spanos et al. [24]	237 (96.3)	324 (93.6)	293 (84.7)	95 (27.5)	52 (77.6)	61 (91)	48 (71.6)	15 (22.4)
Rizwan et al. [25]	114 (77.6)	137 (93.2)	132 (89.8)	40 (27.2)	123 (74.1)	155 (93.4)	159 (95.8)	62 (37.3)

CEA: carotid endarterectomy, CAS: carotid artery stenting, HT: hypertension, DLP: dyslipidemia, DM: diabetes mellitus, NA: not available.

## Supplementary Materials

The following are available online at https://www.mdpi.com/2077-0383/10/5/935/s1, Table S1: P.I.C.O. (patient; intervention; comparison; outcome) model was used to define the clinical questions and clinically relevant evidence in the literature, Table S2: Grading of the retrieved articles with regard to the quality of evidence, Table S3: Summary of evidence, Table S4: The risk of bias of each study included in the analysis was assessed using the Robins I tool for non-randomized trial.
