# Supplementary material for: Early Outcomes of Carotid Revascularization in Retrospective Case Series
Source: J Clin Med. 2021 Mar 1;10(5):935. doi: 10.3390/jcm10050935 (PMC7957582; doi:10.3390/jcm10050935)
Supplement: Supplementary file 1 [file jcm-10-00935-s001.zip › Supplemental FINAL/Supplemental Table S1, PICO.docx]

**Table S1.** P.I.C.O. (patient; intervention; comparison; outcome) model was used to define the clinical questions and clinically relevant evidence in the literature

| **P** | Patient, population or problem | Patients with symptomatic and asymptomatic carotid occlusive disease |
| --- | --- | --- |
| **I** | Intervention, prognostic factor or exposure | CAS and CEA in patients with carotid stenosis in centers providing both treatment modalities |
| **C** | Comparison of intervention | New neurological events, myocardial infarction and composite outcome of stroke/MI and death before and after CAS or CEA |
| **O** | Outcome you would like to measure or achieve | Neurological events (TIAs, stroke), MI and composite outcome of neurological event/myocardial infarction and death in early follow-up after CAS and CEA |
|  | What type of question are you asking? | Are CAS and CEA equally effective and safe in the short-term follow-up when provided in high-experienced single centers? |
|  | Type of study you want to find | Single center prospective or retrospective cohort studies comparing CAS and CEA outcomes in centers providing both techniques |
| CAS: carotid artery stenting; CEA: carotid endarterectomy; MI: myocardial infarction; TIA: transient ischemic attack | | |
