# Supplementary material for: Early Outcomes of Carotid Revascularization in Retrospective Case Series
Source: J Clin Med. 2021 Mar 1;10(5):935. doi: 10.3390/jcm10050935 (PMC7957582; doi:10.3390/jcm10050935)
Supplement: Supplementary file 1 [file jcm-10-00935-s001.zip › Supplemental FINAL/Supplemental Table S2, GRADE.docx]

| **Quality assessment** | | | | | | | | |  |  | |  | |  |  |  |
| --- | --- | --- | --- | --- | --- | --- | --- | --- | --- | --- | --- | --- | --- | --- | --- | --- |
|  |  |  |  |  |  |  |  |  | ***N of patients*** | | ***N of patients*** | | ***Quality*** | | ***Importance*** |  |
| ***No studies*** | | ***Study design*** | ***Risk of bias*** | ***Inconsistency*** | | ***Indirectness*** | ***Imprecision*** | ***Other considerations*** | ***CAS*** | | ***CEA*** | | ***Relative (CI 95%)*** | |  |  |
| *Neurological events during early follow-up;*  *assessed as any neurological event including minor & major stroke and TIAs recorded during the early follow-up* | | | | | | | |  |  | |  | |  | |  |  |
| 13 | | 13 cohort studies | Not serious | Not serious | Not serious | | Serious | None | 4.844 | | 11.428 | | 0.79 | | Moderate xooo | Critical |
| *Myocardial Infarction;, assessed as any myocardial ischemic event during the early follow-up* | | | | | | | |  |  | |  | |  | |  |  |
| 12 | 12 cohort studies | | Not serious | Not serious | Not serious | | Not Serious | None | 4.777 | | 11.082 | | 0.94 | | High  oooo | Important |
| *Composite of neurological event/myocardial infarction and death; assessed as any adverse event recorded during the early follow-up* | | | | | | |  |  |  | |  | |  | |  |  |
| 12 | 12 cohort studies | | Not serious | Not serious | Not serious | | Not serious | None | 4.777 | | 11.082 | | 1.00 | | High  oooo | Important |

**Table S2.** Grading of the retrieved articles with regard to the quality of evidence
