# Supplementary material for: Early Outcomes of Carotid Revascularization in Retrospective Case Series
Source: J Clin Med. 2021 Mar 1;10(5):935. doi: 10.3390/jcm10050935 (PMC7957582; doi:10.3390/jcm10050935)
Supplement: Supplementary file 1 [file jcm-10-00935-s001.zip › Supplemental FINAL/Supplementary Table S4.docx]

| **Studies** | **Confounding** | **Selection bias** | **Classification of intervention** | **Intended intervention** | **Missing data** | **Measurement of outcomes** | **Reported results** | **Overall** |
| --- | --- | --- | --- | --- | --- | --- | --- | --- |
| **Kastrup et al** | Moderate | Low | Moderate | Low | Low | Serious | Low | Moderate |
| **Marine et al** | Moderate | Low | Moderate | Low | Low | Serious | Low | Moderate |
| **Tang et al** | Serious | Moderate | Moderate | Moderate | Low | Serious | Low | Serious |
| **De Rango et al** | Moderate | Low | Moderate | Low | Low | Serious | Low | Moderate |
| **Lindström et al** | Serious | Moderate | Moderate | Moderate | Low | Serious | Low | Serious |
| **Steinbauer et al** | Moderate | Moderate | Moderate | Low | Serious | Serious | Serious | Serious |
| **Tas et al** | Moderate | Low | Moderate | Low | Low | Serious | Low | Moderate |
| **Setacci et al** | Moderate | Low | Moderate | Low | Low | Serious | Low | Moderate |
| **Brooks et al** | Moderate | Moderate | Moderate | Low | Serious | Serious | Serious | Serious |
| **Grimm et al** | Moderate | Low | Moderate | Low | Low | Serious | Low | Moderate |
| **Fantozzi et al** | Serious | Moderate | Moderate | Moderate | Low | Serious | Low | Serious |
| **De Rango et al** | Moderate | Low | Moderate | Low | Low | Serious | Low | Moderate |
| **Meller et al** | Moderate | Low | Moderate | Low | Low | Serious | Low | Moderate |
| **Spanos et al** | Moderate | Low | Moderate | Low | Serious | Serious | Serious | Serious |
| **Rizwan et al** | Moderate | Low | Moderate | Low | Low | Serious | Low | Moderate |

**Supplementary Table S4.** The risk of bias of each study included in the analysis was assessed using the Robins I tool for non-randomized trial.
