# Supplementary material for: Early Outcomes of Carotid Revascularization in Retrospective Case Series
Source: J Clin Med. 2021 Mar 1;10(5):935. doi: 10.3390/jcm10050935 (PMC7957582; doi:10.3390/jcm10050935)
Supplement: Supplementary file 1 [file jcm-10-00935-s001.zip › Supplemental FINAL/Supplemental Table S3, SoF.doc]

| **Outcomes** | **Number of participants Studies Follow-up** | **Quality of evidence (GRADE)** | **Relative effect (CI 95%)** | **Conclusions** | **Recommendations** |
| --- | --- | --- | --- | --- | --- |
| Post-operative neurological events | 11.428 patients with CEA & 4.844 with CAS  13 studies, Early follow-up | Moderate  xooo | 0.79  (0.56;1.11) | There is a moderate body of moderate quality of evidence that there is a low risk for post-operative neurological events | Further evidence from high-quality studies is needed |
| Post-operative myocardial infarction | 11.082 patients with CEA and 4.777 with CAS  12 studies,  Early follow-up | High  xooo | 0.94  (0.80;1.11) | There is a moderate body of high quality of evidence that there is a low post-operative myocardial infarction rate | Further evidence from high-quality studies is needed |
| Composite outcome of neurological event/myocardial infarction/death | 11.082 patients with CEA and 4.777 with CAS  12 studies,  Early follow-up | High  xooo | 1.00 (0.82;1.21) | There is a moderate body of high quality of evidence that there is a low post-operative composite outcome rate | Further evidence from high-quality studies is needed |

**Table S3.** Summary of evidence. CAS: carotid artery stenting; CEA: carotid endarterectomy
